# Anti-Adhesive Podocalyxin Expression Is Disrupted in Recurrent Implantation Failure

**DOI:** 10.3390/diagnostics15010100

**Published:** 2025-01-03

**Authors:** Mustafa Tas

**Affiliations:** Department of Obstetrics and Gynecology, IVF-Unit, Acibadem Kayseri Hospital, 38140 Kayseri, Türkiye; drmustafatas@yahoo.com

**Keywords:** recurrent implantation failure, endometrium, receptivity, podocalyxin, HOXA

## Abstract

**Objectives:** The downregulation of anti-adhesive regulatory proteins and upregulation of adhesive genes are critical for the receptive endometrium. This study was designed to determine whether switching between the anti-adhesive podocalyxin (PDX) and adhesive HOXA10 receptivity modulator occurs in the endometrium of women with recurrent implantation failure (RIF). **Methods:** Twenty-four patients with RIF who could not conceive for three or more cycles despite good-quality embryo transfer constituted the study group. Twenty-four patients with unexplained infertility (UEI) matched for age, BMI, and infertility duration were included in the control group. Twenty women scheduled to undergo intrauterine device (IUD) placement for birth control were included in the comparative group. Endometrial tissue was collected from patients with RIF and UEI during egg retrieval after ovarian stimulation using the GnRH antagonist protocol. In the fertile group, endometrial tissues were collected during IUD insertion. HOXA10 mRNA expression was analyzed by qRT-PCR and PDX protein expression was analyzed by ELISA. The relative expression of endometrial HOXA10 mRNA was calculated using the 2^−ΔΔCt^ equation. **Results:** The relative expression of HOXA10 mRNA in the RIF group was significantly lower than that in the UEI group (*p* < 0.001). HOXA10 mRNA expression in the fertile group was significantly higher than that in the RIF group and was similar to that in the UEI group. PDX expression in the RIF group was significantly higher than that in the UEI group (*p* < 0.001). PDX expression in the fertile group was significantly lower than in the RIF and UEI groups. A negative correlation was detected between the anti-adhesive PDX protein and adhesive HOXA10 (r = −0.797, *p* < 0.001). Although there was a positive correlation between endometrial thickness recorded on the day of hCG administration and HOXA10 (r = 0.590, *p* < 0.01), endometrial thickness was negatively correlated with PDX (r = −0.529, *p* < 0.01). **Conclusions:** The failed physiological downregulation of the anti-adhesive PDX protein in patients with RIF prevented the upregulation of adhesive HOXA10 mRNA.

## 1. Introduction

Implantation failure describes situations in which hCG levels do not increase or the gestational sac does not form despite the hCG increase [1]. Although there is no consensus definition for recurrent implantation failure (RIF), it is defined as the failure to achieve pregnancy in two or three unsuccessful assisted pregnancy cycles despite good-quality embryo transfer [2]. Although the etiology of RIF is unclear, embryo quality, maternal age, and the endometrium are important determinants [3]. There is no standard approach for the diagnosis and treatment of RIF, which is seen in approximately 10% of couples undergoing IVF/ICSI. The etiology of RIF is multifactorial, and anatomical and molecular mechanism disorders of the woman and the man are at the forefront. A limited number of studies investigating the role of the endometrium in the formation of RIF have reported increases in uterine natural killer cell activity [4], the Th1/Th2 ratio [5] and autoimmune antibody levels [6]. Reports of decreased expression of receptivity modulators, such as homeobox transcripts and leukemia inhibitor factor, in some RIF patients have focused on the effects of adhesive and anti-adhesive receptivity modulators in RIF [7,8,9].

The first area, where the embryo connects to the endometrium during implantation, is the luminal epithelium. In the preceptive phase, the luminal epithelium is covered with an anti-adhesive protein with a sialomucin structure, called podocalyxin (PDX). PDX, a member of the CD34 family, is mainly synthesized in the renal glomeruli but is also expressed in the vascular endothelium and many hematopoietic cells [10,11,12]. The endometrium is another important tissue in which PDX is synthesized in the reproductive tract. PDX contributes to the regulation of embryonic development via adaptor proteins. PDX, whose effect in the endometrium is controlled by progesterone, regulates the inflammatory response and keeps the endometrium refractory to implantation until the mid-luteal phase [12]. It is the only known negative regulator protein of embryo attachment and does not allow embryo adhesion to endometrial epithelial cells during the follicular phase [10,11].

Following ovulation, increasing progesterone levels physiologically inhibit PDX production and shift the endometrium to the receptive phase. Luminal PDX expression, which begins to decrease in the early luteal phase, decreases completely in the mid-luteal phase, allowing embryo adhesion [12]. The decrease in PDX expression is accompanied by an increase in the expression of HOXA10, a strong adhesive transcript that opens the implantation window [10].

Because the overexpression of PDX causes functional defects in many tissues, the inadequate downregulation of this molecule may be responsible for failed implantation in patients with RIF. The decreased expression of adhesive receptivity modulators such as LIF and HOXA in RIF patients [7,8] may be due to the downregulation defect in the anti-adhesive PDX protein. HOXA10 is a member of the Abdominal B gene family and is the main adhesive gene responsible for embryo implantation [13]. The HOXA10 transcript, whose expression reaches its maximum in the mid-luteal phase, replaces anti-adhesive receptivity modulators [14,15]. The homozygous mutation of HOXA10 does not affect ovarian reserve but often results in infertility. HOXA10 deletion allows early implantation, but embryos die by apoptosis within a short period [13,14,15]. To date, no study has been conducted to determine whether PDX expression is altered in the endometrium of patients with RIF. Therefore, this study aimed to evaluate whether the physiological transition between adhesive and anti-adhesive receptivity modulators is impaired in patients with RIF. Since the inadequate downregulation of anti-adhesive PDX limits adhesive HOXA10 gene expression, we analyzed the expression levels of these two molecules in endometrial samples from patients with RIF. PDX protein and HOXA10 mRNA expression in mid-luteal phase endometrial tissue samples of RIF patients were compared with those of unexplained infertile and fertile controls.

## 2. Materials and Methods

This cross-sectional study was conducted on infertile patients with a normal ovarian response at the Kayseri Acıbadem Hospital IVF Unit. The participant population was selected from patients who were followed up with a diagnosis of RIF or UEI. After institutional ethics committee approval and consent were obtained (Ethics Approval Number: 2024/11), patient recruitment and sample collection procedures were initiated. After the patients were informed about this study, their consent for endometrial sampling and total embryo freezing was obtained. Patients who did not undergo total freezing or endometrial sampling were excluded from this study, even if they met the inclusion criteria. In the sample size estimation based on the study by Yang et al. [7], the minimum number of samples required to compare three independent groups was determined to be 22 for each group when the effect size f = 0.54, α = 0.01 and power (1 − β) = 90% were accepted. Sample size estimation was performed using G*Power (version 3.1.9.4; Heinrich-Heine-Universität Düsseldorf, Düsseldorf, Germany). Twenty-four patients scheduled to undergo IVF/ICSI and total embryo freezing due to recurrent implantation failure (RIF) constituted the study group. A history of three or more failed IVF/ICSI was considered to represent RIF. Twenty-four patients diagnosed with unexplained infertility (UEI) who were scheduled for IVF/ICSI constituted the comparative group. Women in whom a clear cause of infertility could not be determined by the current methods were diagnosed with UEI. Twenty patients who were scheduled to undergo intrauterine device (IUD) insertion were considered as the fertile control group. Patients in the RIF and UEI groups were matched according to age, BMI and infertility duration.

The inclusion criteria were as follows: 20–35 years of age, normal ovarian response and endometrial anatomy, and no history of endometrial surgery due to polyps or fibroids. The retrieval of 6–14 oocytes was considered a normal ovarian response. The inclusion criteria were the same for both RIF and UEI groups.

After recording the age, body mass index, infertility duration and the number of unsuccessful attempts of all participants, the basal hormone levels were measured on the third day of the cycle. Endometrial thickness (EMT) was recorded by measuring the maximum echogenic length between the endometrium and the myometrium. The antral follicle count was measured by transvaginal ultrasonography. Serum luteinizing hormone (LH), follicular stimulating hormone (FSH) and estradiol levels were measured using an electrochemiluminescent immunoassay. Patients with a history of endometrial polyps, uterine fibroids, mechanical endometrial injury, endometrial surgery, ovarian endometrioma, unilateral or bilateral hydrosalpinx, adenomyosis, PCOS, premature ovarian aging or chronic endometritis were excluded from this study. Patients with systemic inflammatory disease and renovascular pathology, as well as those regularly using vitamin D, nonsteroidal anti-inflammatory drugs and hormonal drugs, were not included in this study.

### 2.1. Ovarian Stimulation and Endometrial Sampling

Participants in the RIF and UEI groups underwent controlled ovarian stimulation using a GnRH antagonist protocol. Recombinant FSH was initiated on day 2 or 3 of the spontaneous menstrual cycle. The starting dose of rFSH was determined on the basis of the patient’s age, AFC and previous cycle responses. Pituitary suppression with a GnRH antagonist was performed when the dominant follicle measured ≥14 mm. Ovulation was triggered by a GnRH agonist when two or more follicles were ≥18 mm in diameter. Endometrial sampling was performed while the patient was under anesthesia immediately after oocyte retrieval, which was performed 36 h after ovulation was triggered. Endometrial tissue samples were collected from the fertile control group by using a Pipelle cannula at the time of tubal ligation or intrauterine device insertion. In the fertile group, endometrial sampling was performed in the early luteal phase, following a negative pregnancy test. Tissue samples were stored at −20 °C in RNA stabilization buffer (RNA-later) until used for qRT-PCR and ELISA analysis. Following ICSI, all the embryos were frozen by vitrification.

### 2.2. Quantitative RT-PCR Analysis

The mRNA expression of HOXA10 was analyzed by qRT-PCR. HOXA10 was preferred for the PCR analysis because it is one of the main adhesive genes responsible for receptivity.

### 2.3. Total RNA Isolation

After the homogenization of the endometrial samples with a tissuelyser, RNA isolation was performed using the PureLink RNA Mini Kit (Thermo Fisher Scientific, Waltham, MA, USA). DNA contamination was prevented using the PureLink DNase kit, and RNA quality was assessed by agarose gel electrophoresis.

### 2.4. Complementary DNA Amplification and HOXA10 mRNA Analysis

Complementary DNA (cDNA) required for HOXA10 mRNA expression was synthesized using a reverse transcription kit in 10 µL RNA samples taken from RNA pools of all three groups. cDNAs was amplified using sequence-specific primers. The primer sequences designed for HOXA10 RT-PCR were forward 5′-GGT TTGTTC TGA CTT TTTGTT TCT-3 and reverse 5′-TGACAC TTA GGACAATAT CTATCTCTA-3, and the housekeeping gene GAPDH sequences were forward 5′- GAA GGT GAA GGT CGG AGT C-3 and reverse 5′-GAA GAT GGT GAT GGG ATT TC-3. mRNA analysis was performed using an amplifyme system (BLIRT S.A., Gdańsk, Poland) and step one plus (Applied Biosystems, Waltham, MA, USA).

### 2.5. Endometrial PDX Analysis with ELISA

PDX is a sialomucin protein with three epitopes. It can be detected using PCR, ELISA and Western blotting. We preferred to analyze with ELISA because of epitope differences and easy accessibility. Endometrial samples from each participant were diluted 1/10 with PBS solution and homogenized in a homogenizer at 15,000 rpm for 10 min, and the supernatant was transferred to Eppendorf tubes and stored at −20 °C. The endometrial PDX concentration was measured using the quantitative sandwich enzyme immunoassay principle in accordance with the manufacturer’s instructions (Sunred Biotechnology Company, Shanghai, China). The measurement range of the PDX kit was 0.2 ng/mL–60 ng/mL, and the minimum measurable level was 0.153 ng/mL. The intra- and inter-assay CV values for the PDX kit were <10% and <12%, respectively. While the Biochrom Anthos Fluido 2 device was used for washing, the absorbances were analyzed using a CLARIOstar PLUS device at a wavelength of 450 nm. The concentration corresponding to the absorbance was calculated using a standard curve formula.

### 2.6. Statistical Analysis

Statistical analysis of the data was performed with SPSS version 27.0 (IBM Corp., Armonk, NY, USA), and GraphPad Prism 8.0 (GraphPad Software, San Diego, CA, USA) programs were used for statistical analysis. The suitability of the data for normal distribution was evaluated using the Shapiro–Wilk test. Data with normal distribution are presented as mean ± standard deviation, and data with non-normal distribution are presented as median (25th and 75th percentiles). Student’s *t*-test was used to compare normally distributed data between two groups, and one-way analysis of variance was used to compare three groups. If the variances of the groups were homogeneous, the Bonferroni test was used to determine the group causing the difference. If they were not homogeneous, Tamhane’s T2 test was used. The Mann–Whitney U test was used for comparisons between two groups of parameters that did not show normal distributions, and the Kruskal–Wallis test was used for comparisons between three groups. The Bonferroni test was used to determine differences between the groups. Spearman’s correlation analysis was used to determine the relationship between the parameters. The Metan package in R was used to create the correlation matrix [16]. Statistical significance was set at *p* < 0.05. The relative expressions of the HOXA10 gene was calculated with the comparative ΔCt method (Ct of target gene—Ct of reference gene) using the following formula: Relative expression = 2^−ΔΔCt^.

## 3. Results

The overall demographic and laboratory data and endometrial PDX and HOXA10 values of the patients in each group are presented in Table 1. In this table, some data specific to infertility patients are highlighted as not applicable (NA) to fertile controls. The mean age, BMI, infertility duration and serum FSH, LH and estradiol levels on the third day of the cycle were similar between the RIF and unexplained infertile groups. There was no significant difference between serum estradiol levels and endometrial thickness in the RIF and UEI groups (Table 1). The mean age and BMI of the participants in the RIF, UEI and fertile groups were similar. The AFCs of patients with RIF and UEI were similarly recorded. The AFC calculations were not performed for the control group.

**Figure 1:**
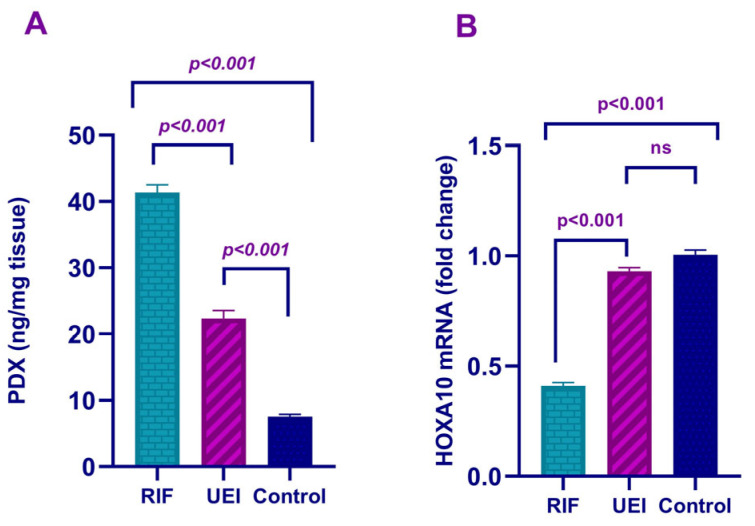
A comparison of anti-adhesive PDX protein (**A**) and adhesive HOXA10 mRNA (**B**) expression in the RIF, UEI and fertile groups. An increase in PDX expression and a decrease in HOXA10 expression indicated the presence of endometrial dysfunction in women with RIF or UEI.

When the groups were evaluated in terms of HOXA10 mRNA expression, it was noted that the relative expression of HOXA10 mRNA in the RIF group was significantly lower than that in the UEI group (*p* < 0.001). The relative HOXA10 mRNA expression in the fertile control group was significantly higher than that in the RIF group, whereas it was similar to that in the UEI group. In contrast, anti-adhesive PDX protein expression in the RIF group was significantly higher than that in the UEI group (*p* < 0.001). The anti-adhesive PDX protein expression in the fertile control group was significantly lower than that in the RIF and UEI groups (Figure 1).

**Figure 2:**
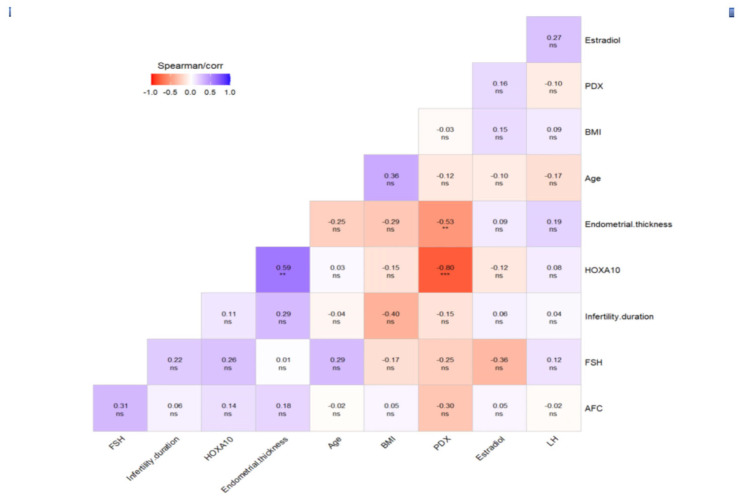
A correlation matrix showing the correlation coefficients between the PDX, HOXA10 and laboratory and demographic variables as a heat map. A correlation coefficient of 0 indicates no correlation between variables, +1 indicates a positive correlation and −1 indicates a negative correlation. ns *p* ≥ 0.05; ** *p* < 0.01; and *** *p* < 0.001.

**Figure 3:**
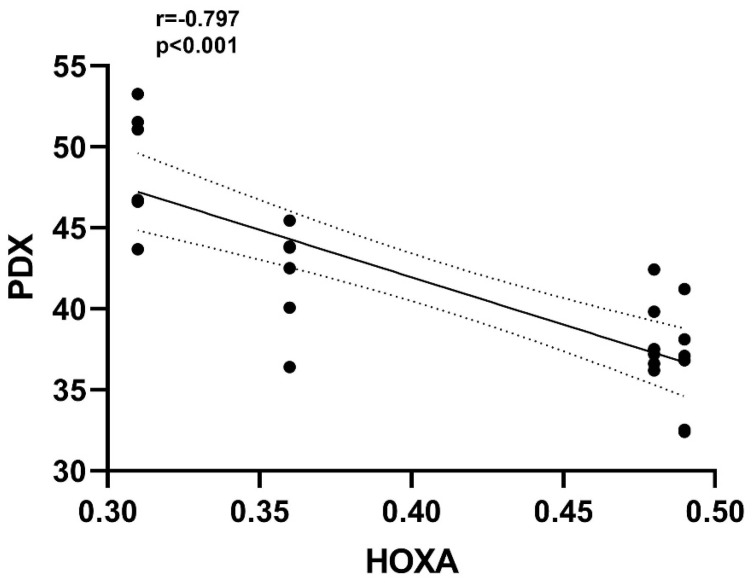
The increase in PDX, accompanied by a decrease in HOXA10, confirms the negative correlation between these two receptivity molecules.

**Figure 4:**
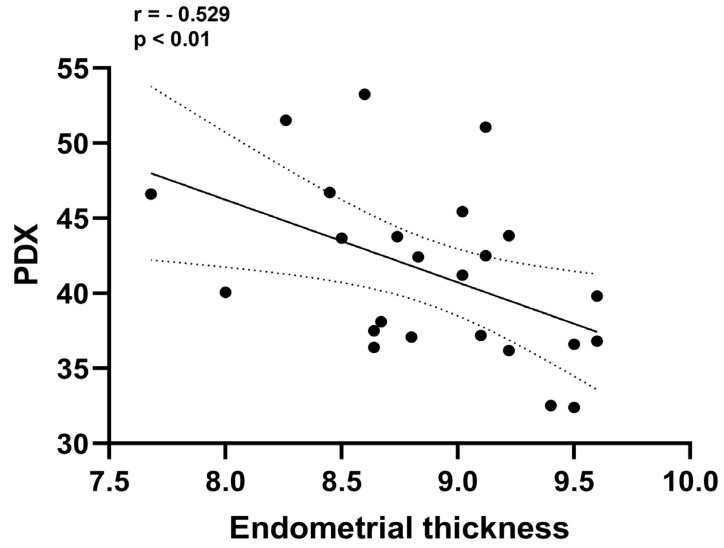
The increase in EMT causes a decrease in PDX expression, confirming that PDX expression is reduced in the presence of a thin EMT.

The correlation matrix shows the correlation coefficients between the PDX, HOXA10 and laboratory and demographic variables as a heat map. A correlation coefficient of 0 indicates no correlation between variables, +1 indicates a positive correlation and −1 indicates a negative correlation. As shown in the correlation matrix (Figure 2), a negative correlation was detected between anti-adhesive PDX protein and adhesive HOXA10 (r = −0.797, *p* < 0.001). Although there was a positive correlation between endometrial thickness recorded on the day of hCG administration and HOXA10 expression (r = 0.590, *p* < 0.01), endometrial thickness was negatively correlated with PDX expression (r = −0.529, *p* < 0.01). Figure 3 shows the negative correlation between the PDX and HOXA10, and Figure 4 shows the negative correlation between the PDX and EMT. The negative correlation between PDX and EMT suggests that an increase in EMT decreases the expression of PDX. The negative correlation between HOXA10 and PDX suggests that adhesive and anti-adhesive genes are negatively affected by the increased expression of each other.

## 4. Discussion

This study is important because it provides the first data showing that anti-adhesive PDX protein expression is defective in the endometrium of patients with RIF. The expression of PDX, which restricts embryo implantation via adaptor proteins and inflammation, was found to be higher in the RIF endometrium than in both patients with UEI and fertile controls. Although the PDX levels of patients with UEI were lower than those of patients with RIF, they exhibited higher PDX expression than that in fertile controls. However, since PDX levels were higher in patients with RIF than in patients with UEI, impaired receptivity was more evident in patients with RIF. In light of these data, we suggest that inadequate PDX downregulation in patients with RIF and UEI contributes to implantation failure.

A competent blastocyst and an endometrium that can perform selectivity and receptivity functions are the two basic elements of successful implantation. Although embryo-related factors are often accepted as the main pathology of recurrent implantation failure, the lack of implantation in 30% of patients who undergo euploid embryo transfer suggests the presence of endometrial dysfunction [17]. Accordingly, a decrease in the expression of HOXA10, a regulatory gene of the homeobox family responsible for embryo adhesion and invasion, has been reported in both RIF and recurrent miscarriage patients [7]. In addition to HOXA10, the detection of a decrease in the expression of E-cadherin, a calcium-dependent cell adhesion molecule, is critical evidence that the balance between adhesive and anti-adhesive regulatory proteins may be impaired in the endometrium of RIF patients [7,18].

Unfortunately, although changes in adhesive genes in RIF have been analyzed, there are no data on anti-adhesive PDX levels in RIF. Podocalyxin is a transmembrane sialomucin protein secreted predominantly by the luminal epithelium and is the only known negative regulator of receptivity [10]. Since a high PDX blocks the opening of the implantation window, adequate expression of adhesive molecules is prevented [11]. The reason for the decrease in the expression of adhesive genes such as HOXA10 in RIF patients may be insufficient downregulation of the PDX protein. The present study is important because it provides the first data showing that the physiological downregulation of PDX expression does not occur in RIF patients. PDX expression of patients in the RIF group was significantly higher compared to the unexplained infertility group, which is another common cause of infertility. Similarly, patients with RIF showed higher PDX expression than fertile controls. Increased PDX expression in RIF patients may be the reason for the decrease in HOXA10 mRNA relative expression. The negative correlation between the anti-adhesive PDX protein and adhesive HOXA10 supports this finding.

The increase in the polarity of Ishikawa cells, which form the receptive endometrial epithelial cell line, in the presence of PDX is evidence that this protein maintains the endometrium in a non-receptive state [10]. It has also been shown that both embryo attachment and invasion are prevented in Ishikawa monolayers exposed to high PDX [18]. The fact that the PDX protein expression of the participants in the RIF group was approximately eight times higher than that of fertile patients and two times higher than that of patients in the EUI group is important evidence that RIF prevents the physiological downregulation of PDX. The fact that HOXA10 mRNA expression was higher in the UEI group, where PDX was low, indicates that PDX inhibits HOXA10 expression. Supporting this, multivariate analysis after adjusting for potential confounders, such as age and number of retrieved oocytes, confirmed that each nanogram increase in PDX increased the risk of HOXA10 mRNA downregulation by approximately 60%.

We do not have clear data regarding the mechanism that causes high anti-adhesive PDX protein expression in patients with RIF. Under normal conditions, luminal epithelial, glandular and endothelial cells of the non-receptive endometrium intensely express PDX [10,11,19]. In the mid-luteal phase, PDX expression begins to disappear, especially on the apical surface of the luminal epithelial cells. However, PDX is also expressed in glandular and vascular endothelial cells. PDX downregulation in apical cells of the luminal epithelium is mediated by progesterone. However, the fact that PDX expression continues in endometrial glands and endothelial cells despite an increase in progesterone is evidence that progesterone selectively expresses PDX only in surface epithelial cells. Since RIF is not an ovulatory pathology [20], it is not reasonable to assume that PDX is not downregulated because of insufficient progesterone production. Since the oocyte retrieval day when endometrial sampling was performed was in the early luteal phase, PDX downregulation may not have occurred because it was still too early for the physiological progesterone peak. However, although UEI is not an endocrine pathology, the fact that PDX levels are lower than those in the RIF group suggests that RIF patients have a special pathology in their endometrium that prevents PDX downregulation. Insufficient progesterone production or progesterone receptor resistance may be the two possible causes of PDX persistence. Studies comparing serum progesterone levels, endometrial progesterone receptors and PDX values on the day of egg retrieval may clarify this issue.

The negative correlation between endometrial thickness and PDX expression and a positive correlation with HOXA10 expression suggest that the endometrium must have reached sufficient thickness for the critical balance between anti-adhesive and adhesive receptivity regulators [21]. Although the endometrial thicknesses of the RIF and EUI groups were similar, the persistence of PDX expression in the RIF group suggests that a process known as epithelial-mesenchymal transition, which enables the transition of the endometrium from a non-receptive state to a receptive state, does not occur in RIF [22,23]. Moreover, PDX overexpression may prevent implantation by disrupting the solid-state endometrial signaling pathway required for implantation [24].

Despite the small number of participants, our results are of critical importance in demonstrating that physiological downregulation of the anti-adhesive PDX protein does not occur in RIF patients. More participants would have helped to detect rarer differences and helped us to have a clearer discussion. However, because the number of participants determined by power analysis is sufficient for statistical analysis, our results can be generalized. The inadequate downregulation of PDX prevents the opening of the implantation (WOI) and restricts embryo adhesion. Similar to RIF, PDX was inadequately downregulated in patients with UEI. In contrast, adhesive HOXA10 expression was decreased in both the RIF and UEI patient groups. The increase in PDX in the RIF group was more pronounced than that in both the UEI and control groups. The increase in PDX expression may be the main reason for the decrease in HOXA10 expression in the RIF group. The PDX level in the UEI group was higher than that in the control group but half that in the RIF group. The HOXA10 level in the UEI group was approximately twice that of the RIF group. In light of these data, we can attribute the low HOXA10 expression in the RIF group to the insufficient downregulation of PDX expression.

Two studies conducted in 2021 have shown that podocalyxin is a major negative regulator of human embryo implantation [10,11]. PDX is thought to exert its in vitro embryo implantation-reducing effect by reducing the expression of adhesive molecules such as LIF [25]. However, there is only one paper in the literature that clearly demonstrates the relationship between PDX and the adhesive HOXA genes. The negative correlation between PDX and HOX10 in RIF patients supports the idea that PDX inhibits HOXA10 expression.

Although it is the first to show endometrial PDX expression in RIF patients, this study had some limitations. Although the number of participants was sufficient to detect basic differences between the groups, more participants would have been useful to detect rarer differences. Although preimplantation genetic diagnosis (PGD) applications for achieving healthy pregnancies in RIF patients have recently attracted attention, concerns still persist due to problems such as polyploidy and somatic mosaicism [26]. Another limitation is the lack of PGD results in most of our participants.

This study was the first to perform endometrial PDX analysis in RIF patients. The participant cohort consisting of three different patient groups allowed for the generalization of the obtained data. The combination of high PDX and low HOXA10 expression may be one of the molecular reasons for unsuccessful implantation in patients with RIF and UEI. HOXA10 and PDX analyses can be performed in RIF patients to determine whether WOI is open. Because of the negative correlation between PDX and EMT, clinicians may postpone embryo transfer, considering that PDX expression is high in the presence of subtle EMT. The inclusion of HOXA10 and PDX in the endometrial receptivity array (ERA) test set may increase the reliability of the ERA test. The need for endometrial sampling for PDX and HOXA assessments may be an invasive limitation on the development and use of this test. Comprehensive studies on the development of rapid and practical tests to analyze these two molecules in endometrial secretomes or cervical mucus may lead to a new era in reproductive biology.

## Figures and Tables

**Table 1:** A comparison of the demographic characteristics of the RIF, UEI and fertile groups.

	RIF Group (*n* = 24)	UEI Group (*n* = 24)	Fertile Group (*n* = 20)	*p*-Value
Age (years)	29.6 ± 3.82	28.9 ± 2.39	30.4 ± 2.76	0.311 ***
BMI (kg/m^2^)	23.10 ± 1.35	23.34 ± 1.55	23.22 ± 1.81	0.876 ***
Infertility duration (yrs)	4 (3–5)	4 (3–4)	NA	0.661 **
Antral follicle count	9 (9–10)	9.5 (9–10)	NA	0.277 **
Estradiol (pg/mL)	38.30 ± 10.78	40.39 ± 9.66	45.12 ± 11.62	0.108 ***
LH (mIU/mL)	5.58 ± 1.18	5.99 ± 1.57	6.14 ± 1.58	0.398 ***
FSH (mIU/mL)	5.34 (5.25–5.39)	5.45 (4.9–5.6)	5.49 (5.27–5.64)	0.145 ****
EMT on the day of hCG (mm)	9.4 ± 0.5	9.6 ± 0.8	NA	0.306 *
E2 on the day of hCG (pg/mL)	1637.13 ± 330.07	1788.94 ± 349.17	NA	0.129 *
PDX (ng/mg)	41.37 ± 5.71 ^a b^	22.33 ± 5.96 ^a^	7.57 ± 1.40	<0.001 ***
HOXA10 mRNA	0.41 ± 0.07 ^a b^	0.93 ± 0.08	1.01 ± 0.09	<0.001 ***

* Student’s *t*-test, ** Mann–Whitney U test, *** One-Way Anova test, **** Kruskal–Wallis test. ^a^ *p* < 0.001 vs. fertile group; ^b^ *p* < 0.001 vs. *UEI group*. RIF: recurrent implantation failure; UEI: unexplained infertility; NA: not applicable; EMT: endometrial thickness.

## Data Availability

All datasets used and/or analyzed during the current study are available in this study, and there are no additional data.
